# MiR-223 Suppresses Cell Proliferation by Targeting IGF-1R

**DOI:** 10.1371/journal.pone.0027008

**Published:** 2011-11-02

**Authors:** Cheng You Jia, Hui Hui Li, Xu Chao Zhu, Yi Wei Dong, Da Fu, Qian Lei Zhao, Wei Wu, Xing Zhong Wu

**Affiliations:** Department of Biochemistry and Molecular Biology, Shanghai Medical College, Fudan University, Key Lab of Glycoconjugate Research, Ministry of Public Health, Shanghai, China; Roswell Park Cancer Institute, United States of America

## Abstract

To study the roles of microRNA-223 (miR-223) in regulation of cell growth, we established a miR-223 over-expression model in HeLa cells infected with miR-223 by Lentivirus pLL3.7 system. We observed in this model that miR-223 significantly suppressed the proliferation, growth rate, colony formation of HeLa cells *in vitro,* and *in vivo* tumorigenicity or tumor formation in nude mice. To investigate the mechanisms involved, we scanned and examined the potential and putative target molecules of miR-223 by informatics, quantitative PCR and Western blot, and found that insulin-like growth factor-1 receptor (IGF-1R) was the functional target of miR-223 inhibition of cell proliferation. Targeting IGF-1R by miR-223 was not only seen in HeLa cells, but also in leukemia and hepatoma cells. The downstream pathway, Akt/mTOR/p70S6K, to which the signal was mediated by IGF-1R, was inhibited as well. The relative luciferase activity of the reporter containing wild-type 3′UTR(3′untranslated region) of IGF-1R was significantly suppressed, but the mutant not. Silence of IGF-1R expression by vector-based short hairpin RNA resulted in the similar inhibition with miR-223. Contrarily, rescued IGF-1R expression in the cells that over-expressed miR-223, reversed the inhibition caused by miR-223 via introducing IGF-1R cDNA that didn't contain the 3′UTR. Meanwhile, we also noted that miR-223 targeted Rasa1, but the downstream molecules mediated by Rasa1 was neither targeted nor regulated. Therefore we believed that IGF-1R was the functional target for miR-223 suppression of cell proliferation and its downstream PI3K/Akt/mTOR/p70S6K pathway suppressed by miR-223 was by targeting IGF-1R.

## Introduction

MicroRNAs (miRNAs) are short (20-23 nucleotides), endogenous, single-stranded RNA molecules that regulate gene expression [1], [2]. MicroRNA-223 (miR-223) was identified bioinformatically and subsequently characterized in the hematopoietic system, where it is mainly expressed in the myeloid, granulocytic, and monocytic compartments [3], [4], but not in B and T lymphocytes. The highest levels of expression is observed in bone marrow CD34^-^ fraction, that is representative of lineage-committed precursors and mature hematopoietic cells [5]. The miR-223 locus is located on the X chromosome and is transcribed independently of any known genes [5], [6]. MiR-223 acts as “a fine-tuner” of granulocytic differentiation and maturation [7] and promotes granulocytic differentiation in acute promyelocytic leukemia (APL) cells treated with retinoic acid (RA) which can induce up-regulation of C/EBPα (CCAAT-enhancer-binding proteins α). C/EBP αcan further compete with NF1A and promote miR-223 expression [5], [6]. The expression of miR-223 was then reported to promote granulocytic differentiation [8].

The abnormal signal pathway activation is important in tumor and leukemia cell development. This includes PI3K/Akt, mTOR(mammalian target of rapamycin), ERK/MAPK, STAT3/5, NF-kB, protein kinase C [9], [10] and Wnt/β-catenin [11] as well as insulin-like growth factor-1 receptor (IGF-1R) signal pathway. IGF-1R system is comprised of two ligands (IGF-1,2); three cellular membrane-spanning receptors IGF-1 receptor (IGF-1R), insulin receptor, and IGF-2R; and six high-affinity IGF-binding proteins IGFBP1-6, playing the pivotal role in normal growth and development of the cells [12]. After IGF-1 binding to IGF-1R, the signal pathway PI3K/Akt and mTOR are activated to regulate cell proliferation, and are also activated in tumor cells such as acute myeloid leukemia [11]. Once activated, the signaling through Akt can be propagated to a diverse array of substrates including mTOR, a key regulator of protein translation. This pathway is an attractive therapeutic target in cancer treatment because it serves as a convergence point for many growth stimuli, and through its downstream substrates, it controls cellular processes that contribute to the initiation and maintenance of cancer [13].

However, the detailed mechanisms of miR-223 in differentiation or tumor progression still remain unclear. The functions of miR-223 in previous reports were not clear or somewhat contradicted in both hematopoietic and non-hematopoietic systems. Although miR-223 was thought to promote differentiation, some documents reported that miR-223 negatively regulates granulocyte differentiation in miR-223^-/Y^ transgenic mice [14]. It was also reported that miR-223 was significantly up-regulated in bladder cancer [15] and recurrent ovarian cancer [16]. In hepatocellular carcinoma cells (HCC) miR-223 was repressed as compared with normal liver tissue by microarrays [17] and STMN1 was the potential target which serves as an oncogene implicating that miR-223 may serve as a tumor suppressor[18]. In this study, we investigated the roles of miR-223 in cell growth and sought for the mechanism by which the inhibition of cell growth by miR-223 is mediated.

## Results

### Over-expression of miR-223 in HeLa cells

**Figure 1:**
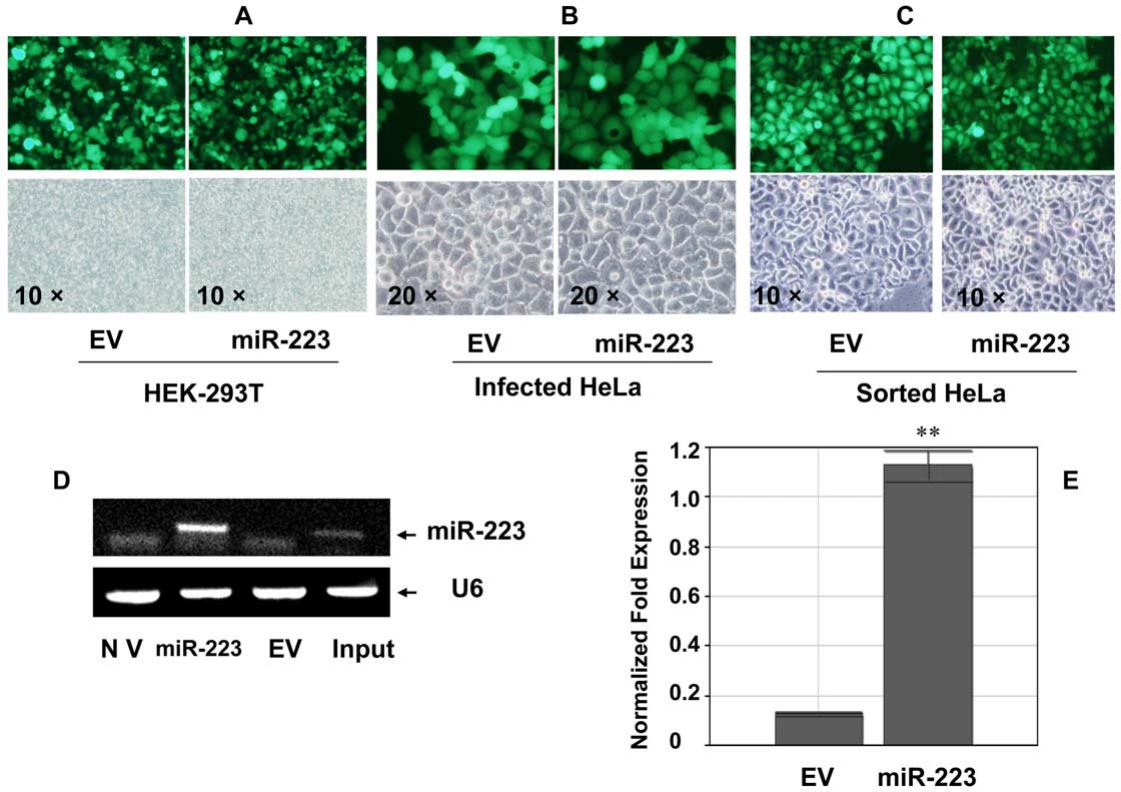
Lentivirus-mediated miR-223 over-expression in HeLa cells. (A) Lentivirus vector pLL3.7-miR-223 was packaged in HEK-293T cells which was less 20 generations. The packaging efficiency was evaluated by GFP fluorescent signal. Original magnification 10 × (B) HeLa cells were infected with the Lentivirus which was packaged and prepared from HEK-293T cells. The GFP fluorescent signal could be stably observed 72 hours after infection. EV group stood for vector control at the left and pLL3.7-miR-223 infection group was at the right. Original magnification 20 × (C) The infected HeLa cells were sorted by FACS. The fluorescence-positive cells were collected in either EV control (left) or miR-223 (right) group. Original magnification 10× (D,E) Mir-223 was over-expressed in HeLa cells and confirmed by both stem-loop RT-PCR (D) and quantitative PCR (E). NV: no virus control, EV: empty virus vector control, Input: positive mature miR-223.

Lentivirus vector is an efficient, stable gene delivery tool in mammalian cells to induce stable gain- and loss-of-function phenotypes for individual miRNAs [19] or shRNAs [20]. Therefore Lentivirus vector pLL3.7 was used in this study to establish miR-223 over-expression. Packaging of virus was performed by co-transfection of three plasmids and the efficiency was about 70% (Fig. 1-A) according to GFP (green fluorescent protein) signal that was carried by pLL3.7 vector or pLL3.7-miR-223. Infection was performed with the lentivirus at a ratio of 1∶1 (v/v) with 8 µg/ml polybrene, twice. After infection of HeLa cells with either EV(Empty Virus Vector) (Fig. 1-B, left) or pLL3.7-miR-223 (right), the infected cells were screened and sorted by a FACS machine based on the expression of GFP which indicated the presence of the plasmids pLL3.7-miR-223 or the EV (Fig. 1-C). The expression of miR-223 in miR-223 group was up-regulated 7.19 folds after sorting as compared with EV group, which was confirmed by both semi-quantitative RT-PCR (reverse transcription polymerase chain reaction) and quantitative PCR (Fig. 1-D, E) besides the fluorescent measurement. The sorted miR-223-infected HeLa cells were then used as the stable miR-223 overexpression model. We referred to the infected cells by either miR-223 or EV as miR-223 or EV groups respectively, in the following experiments.

### MiR-223 suppression of HeLa cell proliferation

**Figure 2:**
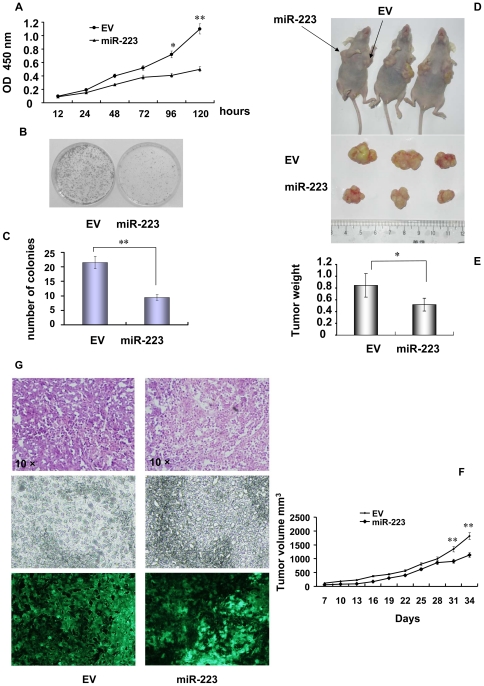
Overexpression of miR-223 suppressed HeLa cell growth. (A) Growth curves of miR-223 and EV-infected HeLa cells were conducted by CCK-8 assay. The OD value at 450 nm represented the viable cell numbers. All experiments were carried 3 times independently. **p<0.01 (B) Colony formation was assayed in miR-223 and EV-infected HeLa cells, and colonies consisting more than 50 cells were counted. The experiment was repeated 3 times independently. (C) Bar graphs show the average number of colonies and comparison between EV and miR-223 groups. **p<0.01 (D) Tumor sizes of 3 representative nude mice. MiR-223(right) and EV group(left) cells were injected subcutaneously in 8 female nude mice. The tumor volume was measured every 3 days with calipers after tumor appeared. (E) Bar graphs show the average tumor weight (gram). * *p*<0.05 (F) Tumor size observation in nude mice after the inoculation. The average size of the tumors was measured on every three days and shown in the curves. The error bars show SD (standard deviation). (G) Histological examination of tumor tissues formed in nude mice. HE (hematoxylin and eosin) staining was at the top panel. The middle panel shows the examination under a phase contrast microscope. The bottom panel shows the GFP signal carried by the pLL3.7 vector under a fluorescent microscope and indicated the formation of tumor caused by either miR-223 or EV-infected HeLa cells.

To observe the effect of miR-223 on the HeLa cells, cell growth rate in stable miR-223-infected HeLa cells was evaluated by CCK-8 assay. In the 5 day's growth rate observation, the proliferation rate of miR-223 group was reduced 2.42 folds as compared with EV group (*p*<0.05) (Fig. 2-A). To further evaluate the proliferation ability, we then performed the colony formation assay and found that the capacity of colony formation was significantly inhibited in MiR-223 group. The number of colonies, which were defined as more than 50 cells which derived from a single cell, in miR-223 group was greatly repressed to 43.72% of the control and the difference of the colony number between the two groups reached significance (*p*<0.01) (Fig. 2-B,C). To further confirm the above findings, an *in vivo* model was carried out by subcutaneous injection of 5×10^6^ EV or miR-223-infected cells into the mouse skin under the front right or left legs respectively. The tumor mass became palpable 8 to 11 days after inoculation in all (8/8) mice in EV group (three representative mice in Fig. 2-D), but no tumor was observed in one of the eight mice in MiR-223 group. Five weeks after inoculation, all mice were sacrificed and the tumor mass was weighted. The average tumor weight of miR-223 group was significantly less than control (*p*<0.05, Fig. 2-E). Tumor size in two groups were measured every three days after tumor were palpable and the results indicated that tumor volumes in miR-223 group only achieved 62.3% of the control (*p*<0.05) (Fig. 2-F). All the tumor masses were examined and confirmed histologically by HE (hematoxylin and eosin) staining (Fig. 2-G). These results suggested that miR-223 suppressed the proliferation of HeLa cells both *in vitro* and *in vivo*.

### IGF-1R was targeted by miR-223

**Figure 3:**
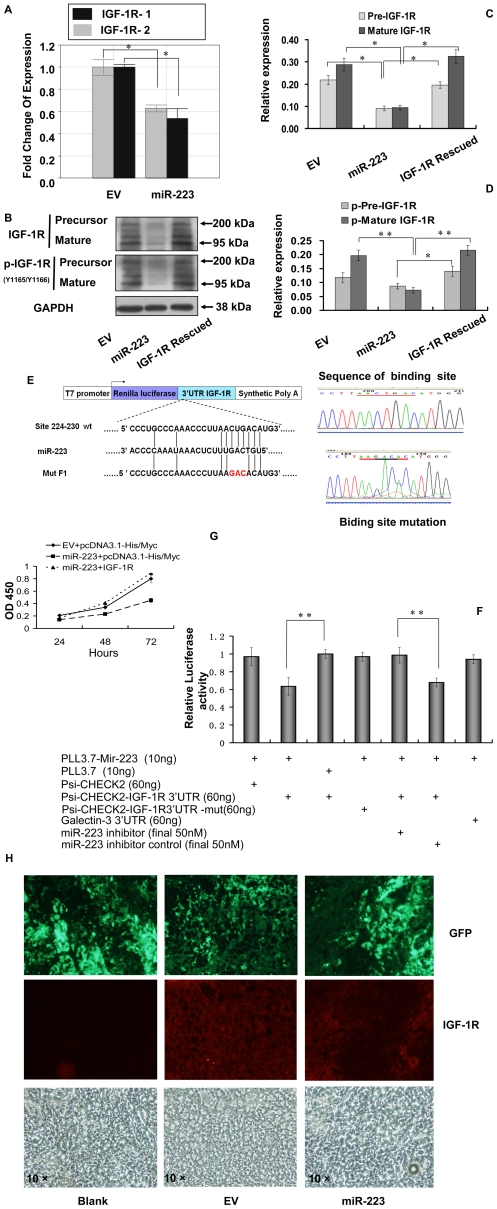
IGF-1R was directly targeted by miR-223. (A) Quantitative PCR results of IGF-1R. Two pairs of primers (IGF-1R-1 and IGF-1R-2) designed at different positions of IGF-1R produced similar results showing suppression caused by miR-223. (B) IGF-1R protein level was suppressed by miR-223. Both precursor and mature IGF-1R (2 bands) were suppressed after miR-223 overexpression. The phosphorylation level was subsequently down regulated in agreement with the total protein level of IGF-1R. The suppression was abolished by transfection of the cells with IGF-1R cDNA without 3′UTR. (C) (D) The protein bands were scanned to measure the integral density in B experiments and normalized to those of GAPDH. The relative expression was then compared. Experiments were carried out 3 independent times. *p<0.05, **p<0.01 (E) The top panel showed the structure and cloning site of psi-*CHECK*™-2 vector. Wild type and three nucleotides mutated within IGF-1R 3′UTR were cloned into the reporter based on the predicted binding site in the 3′UTR of IGF-1R with miR-223 seed sequence. The right panel showed that sequences of either wild type (upper) or mutant (down) were confirmed by sequencing. (F) Dual Luciferase experiment of IGF-1R 3′UTR was conducted. The expression of the reporter containing IGF-1R 3′UTR was suppressed by miR-223, but not in the mutated construct. Inhibition of miR-223 abolished the suppression of miR-223 on IGF-1R 3′UTR at a final concentration of 50 nM. **p<0.01 (G) Growth curve was measured by CCK-8 assay and the results indicated that the suppression of miR-223 group could be overcome by re-expression of IGF-1R. The group transfected with pcDNA 3.1-His/Myc served as the control. **p<0.01 (H): Immunohistochemical staining of IGF-1R in the sections. IGF-1R was labeled in red carried by secondary antibody and the signal was stronger in EV group. In miR-223 group, the signal of IGF-1R staining was significantly weaker than that in EV group. In blank group, PBS replaced the first antibody in either EV or miR-223 group. Original magnification 10 ×.

To further investigate the mechanism of miR-223 inhibition of tumorigenicity and proliferation of HeLa cells, we searched for potential targets by using the prediction algorithm of Targetscan 5.1, 9 molecules including POLE3, POLR3G, FBXW7, IGF-1R, p70S6K, Rasa1, Fox O1, Fox O3 and Cdc27 were selected. Screen results by quantitative PCR suggested that the expression of POLE3, POLR3G, FBXW7, Mef 2C, LMO2, NF1A and STMN1 did not reduce significantly in miR-223 group although the examination was at mRNA levels. However, we found that IGF-1R mRNA was reduced significantly in miR-223 group as compared with EV group (*p*<0.05) (Fig. 3-A). Using 2 different pairs of primers achieved the same results. Precursor and mature IGF-1R proteins were also significantly reduced to 41.4% and 32.7% in miR-223 group as compared with EV group (*p*<0.01) (Fig. 3-B,C). The phosphorylated IGF-1R was reduced accordantly (Fig. 3-B,D). To confirm that IGF-1R was targeted by miR-223, we cloned the 3′UTR of IGF-1R into the site directly downstream of the luciferase reporter gene in psi-*CHECK*™-2 and constructed the three nucleotide mutation within the region that miR-223 seed sequence potentially bound with (Fig. 3-E). We found that the relative luciferase activity of the reporter that contained wild-type 3′UTR of IGF-1R was significantly suppressed in miR-223 group as compared with EV group (*p*<0.01) (Fig. 3-F). However, the relative luciferase activity of the mutant IGF-1R 3′UTR reporter construct with 3 nucleotide mutation within the putative seed sequence (226-228bp in IGF-1R 3′UTR) (Fig. 3-E) was almost at the same level as the control psi-*CHECK*™-2 group and failed to respond to miR-223 (Fig. 3-F). MiR-223 suppression of IGF-1R expression could be reversed by adding exogenous miR-223 inhibitor at the final concentration of 50 nM (*p*<0.01) (Fig. 3-F). By re-expression of IGF-1R, the growth rate of miR-223 group that was transfected with IGF-1R rose after 48 hours and reached to 1.98 folds as much as the group of miR-223 at the time point of 72 hours post transfection (Fig. 3-G). Transfection with IGF-1R cDNA(complementary DNA) could totally overcome the suppression caused by miR-223 (Fig. 3-B) since the construct contained no 3′UTR. In the tumor tissues at xenograft nude mice, the expression of IGF-1R was also suppressed in miR-223 group as compared with EV group (Fig. 3-H).

### MiR-223 inhibition of Akt/mTOR/p70S6K signal pathway

**Figure 4:**
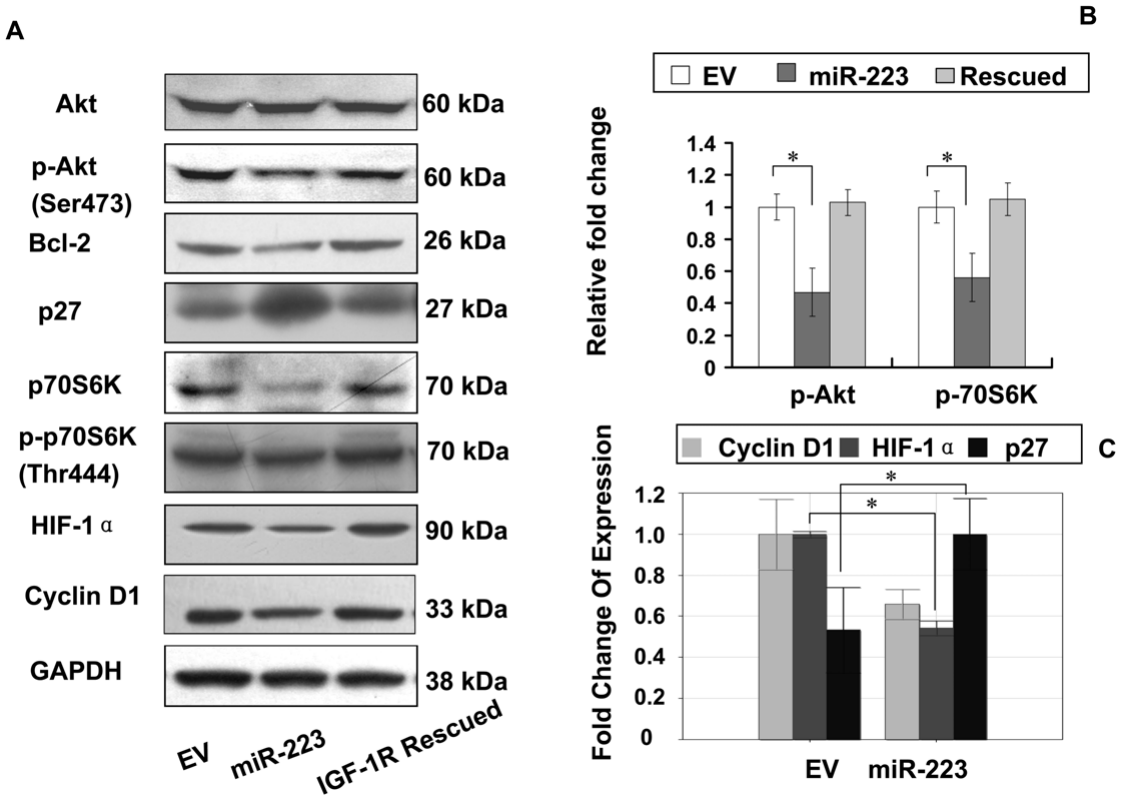
MiR-223 suppressed IGF-1R-mediated Akt/mTOR/p70S6K signal pathway. (A): The Akt/mTOR/p70S6K pathway downstream of IGF-1R were suppressed in miR-223 group. By Western blot, the two major protein kinases Akt and p70S6K revealed significant suppression and were less phosphorylated. P27 protein was up-regulated. Bcl-2, a protein that was promoted by Akt was down-regulated. HIF-1α, the direct target of p70S6K, was significantly inhibited. The inhibition of Akt/mTOR/p70S6K was reversed by re-expression of IGF-1R (IGF-1R rescued) in miR-223 group. (B): Quantification of p-Akt, p-p70S6K in Fig. 4-A by densitometry to analyze the integral density of each band. *p<0.05 (C): Quantitative PCR analysis of the mRNA expression levels of HIF-1α, p27, and Cyclin D1. *p<0.05.

As miR-223 suppressed IGF-1R expression, the next question that needed to uncover was whether the IGF-1R-mediated downstream signal pathway was also impacted by miR-223. To this end, the expressions of Akt, an essential protein kinase in PI3K/Akt pathway downstream of IGF-1R and of its active form (p-Akt) were examined. We observed that the p-Akt was reduced to about 32.4% of the EV group, but the total Akt was unaffected (Fig. 4-A,B). The molecules downstream and inhibited by Akt/p-Akt including p27 were up-regulated (Fig. 4-A). The up-regulation of p27 at mRNA level was further supported by quantitative PCR (Fig. 4-C). In contrast, cyclin D1 and Bcl-2, an anti-apoptotic regulator, that are normally promoted by p-Akt, were down regulated at both protein and mRNA levels (Fig. 4-A,C). To further investigate the pathway alteration at miR-223 group, we detected p-70S6K, a key protein kinase in mTOR signal pathway, and its active form (p-p70S6K) and found that p-p70S6K was greatly reduced in miR-223 group to 58% of the EV group (Fig. 4-A,B), but total p70S6K was unaffected. Since p70S6K is a regulator for HIF-1α expression in endothelial cells, and is required for the cancer cell-induced tumor growth and angiogenesis [21] and for vascular endothelial growth factor (VEGF) expression, we also examined HIF-1α expression in this model to observe the effect of p70S6K inhibition. Interestingly, HIF-1α was strikingly reduced to 54.4% in miR-223 group as compared with control EV group (Fig. 4-A,C), which was consistent with a previous report [21]. Moreover, re-expression of IGF-1R which did not contain the 3′UTR totally reversed the inhibition of Akt/mTOR/p70S6K signal pathway profile (Fig. 4-A).

### Knockdown of IGF-1R mimicked miR-223 inhibition

**Figure 5:**
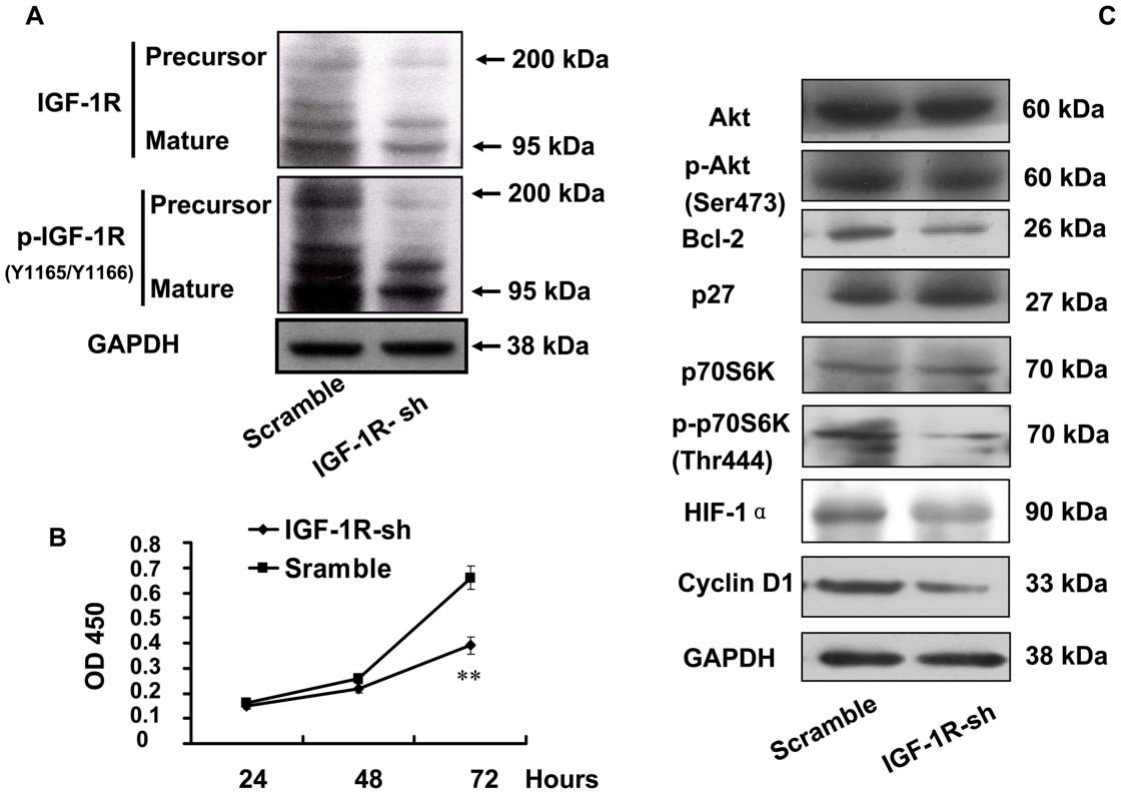
Interference of IGF-1R mimicked the suppression of growth and Akt/mTOR/p70S6K signal pathway by miR-223. (A): Inhibition of IGF-1R was seen after transfection with p*Silencer* 4.1-IGF-1R-shRNA to HeLa cells. Both phosphorylation and total protein levels were reduced and the results were similar to the inhibition miR-223. (B): Growth rate in HeLa cells transfected with IGF-1R-sh was measured by CCK-8 kit. **p<0.01 (C): The Akt/mTOR/p70S6K signal pathway was suppressed by transfection of IGF-1R-sh1, which was similar with the suppression caused by miR-223.

Our results thus far demonstrate that miR-223 suppressed IGF-1R in both mRNA and protein levels, and subsequently suppressed the downstream Akt/mTOR/p70S6K signal pathway. To further demonstrate that IGF-1R targeting by miR-223 is sufficient to effect the reduced cell proliferation phenotype, we performed a loss-of-function experiment by transfection of IGF-1R-sh carried by plasmid p*Silencer 4.1*CMV-puro into HeLa cells. The expression of IGF-1R was successfully knocked down (Fig. 5-A) by IGF-1R-sh through transient transfection, and this led to a similar suppression of the cell growth as miR-223. Knockdown of IGF-1R not only decreased cell viability (Fig. 5-B), but also inhibited the PI3K/Akt/mTOR/p70S6K signal pathway (Fig. 5-C) mentioned above, which was quite similar to the inhibition by miR-223. These results strongly indicated that miR-223 suppressed of Akt/mTOR/p70S6K pathway is by targeting IGF-1R.

### The regulation of miR-223 relied on the pathway triggered by the target

**Figure 6:**
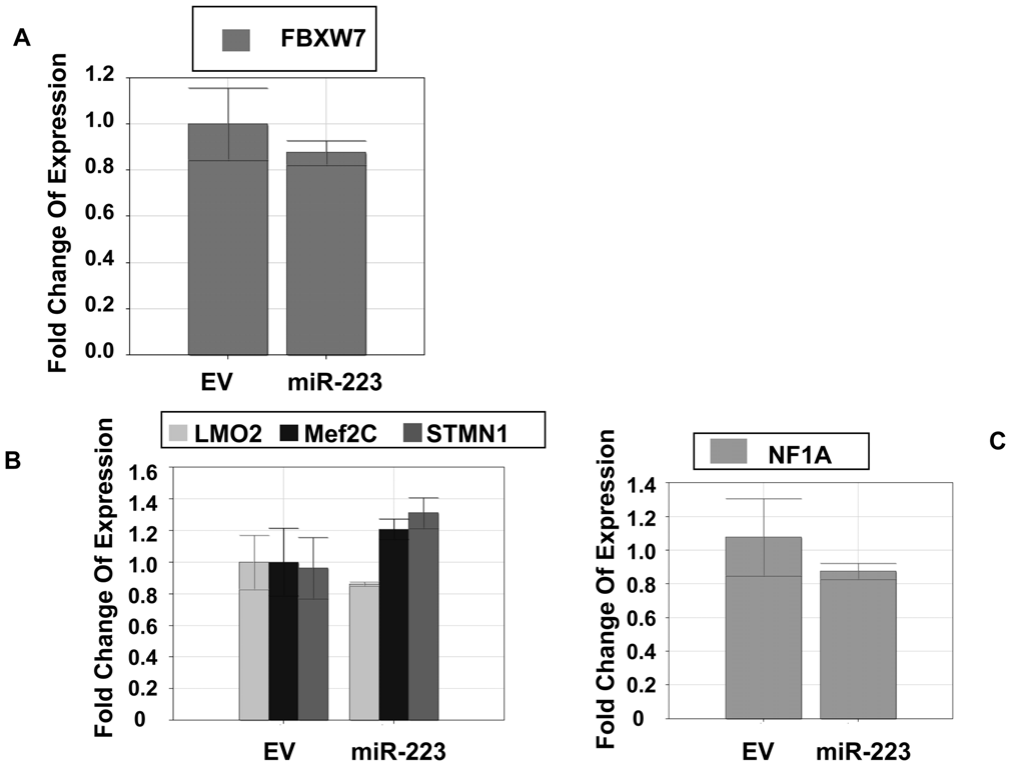
Quantitative PCR analysis of the mRNA expression levels of FBXW7 (A), LMO2, Mef 2C and STMN1 (B), NF1A (C) in EV and miR-223 groups.

**Figure 7:**
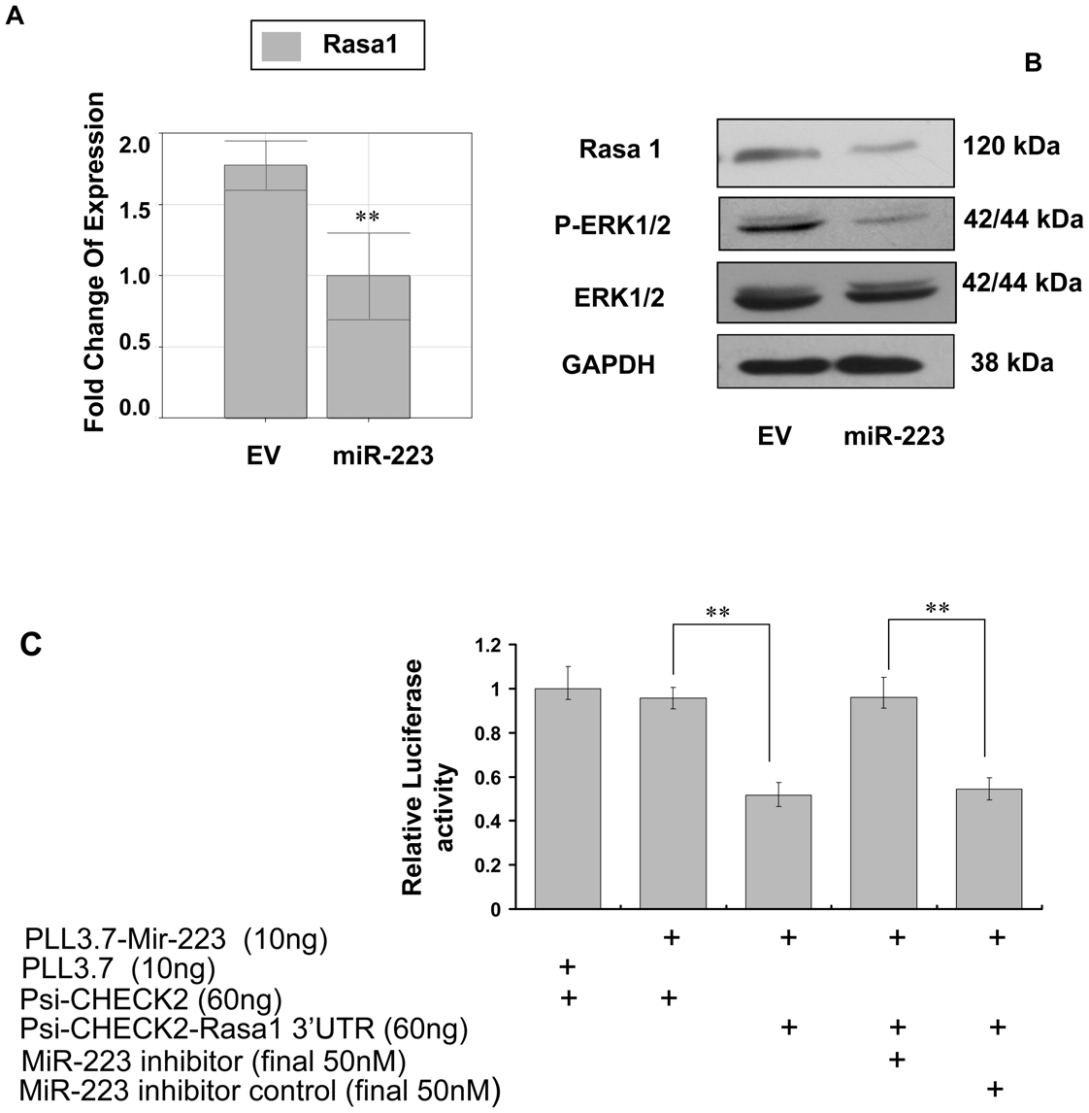
The expression profile of Rasa1/RAF/MEK/ERK pathway. (A): Quantitative PCR analysis of the mRNA levels of Rasa1. (B): Western blot analysis of the protein levels of Rasa1, p-ERK1/2 and ERK1/2. GAPDH served as a loading control. (**C**): Dual luciferase report assay showed that Rasa1 was targeted by miR-223. The expression of Rasa1 was suppressed by miR-223, but the mutant couldn't. The inhibitor of miR-223 abolished the suppression of miR-223 on Rasa1 at a final concentration of 50 nM. Three independent experiments were carried out to achieve similar results. ** *p*<0.01.

Since one miRNA may target a dozen of targets, in this model we further investigated the response of several additional mRNA targets that might be regulated. However, mRNAs predicted by Targetscan 5.1 and reported previously, such as LMO2, STMN1, Mef 2C, FBXW7 and NF1A (Fig. 6), did not significantly decrease in our system although they were examined only at mRNA level. Rasa1 was predicted by Targetscan 5.1 as a target of miR-223 and indeed observed to be targeted by miR-223 in current study at both mRNA and protein levels (Fig. 7-A,B). The luciferase reporter assay did show that the 3′UTR of Rasa1 mRNA was targeted by miR-223 directly (Fig. 7-C). Rasa1 exerted a tumor suppressor function by removing GTP from RAS-GTP. Its down-regulation should activate the Rasa1/RAF/MEK/ERK signal pathway. However, ERK1/2 was down-regulated not only at the total protein level, but also at phosphorylation level in miR-223 group as compared with EV group (Fig. 7-B) which indicated that ERK pathway was inhibited even though Rasa1 was targeted. Therefore Rasa1 could not be the functional target of miR-223 in this system because it failed to regulate ERK pathway after miR-223 targeting. Suppression of IGF-1R-mediated pathway might lead to inhibition of ERK signaling, which may circumvent any regulatory role for Rasa1 in this system. Whether a miRNA can give dominant and full play to the regulation of cells may thus depend on the relative importance of the target that involved in the signal pathway.

### MiR-223 regulation of IGF-1R in leukemia and hepatoma cells

**Figure 8:**
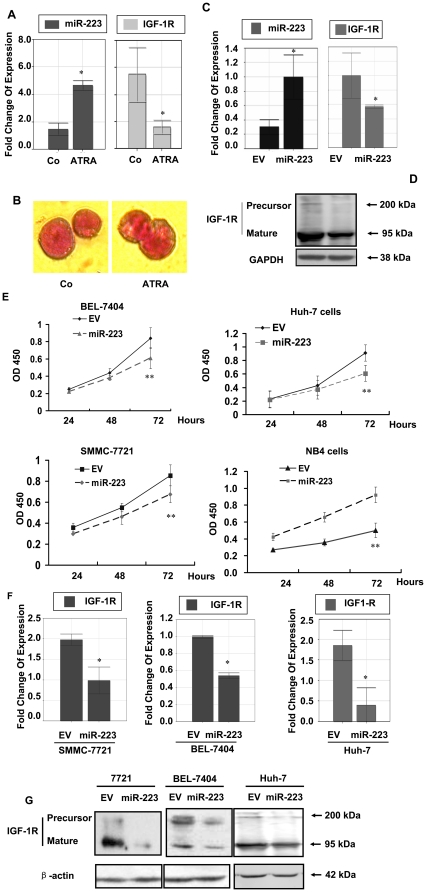
miR-223 regulated IGF-1R in other tumor cells. (A) NB4 cells were treated with 1 µmol/L retinoic acid for 48h and the expression level of miR-223 was determined by quantitative PCR (left panel). In the treated cells the expression level of IGF-1R was measured by quantitative PCR (right panel). (B) Wright-Giemsa staining of the cells. Much more post-mitotic cells (metamyelocytes, segmented neutrophils) were seen in the treated group. Original magnification 100× (C) NB4 cells were infected with miR-223 construct. High expression of miR-223 (left panel) was confirmed by quantitative PCR. The expression level of IGF-1R was also examined and down regulated in miR-223 group. (D) Western blot analysis of IGF-1R expression in NB4 cells infected with miR-223 construct. (E) Growth curve of BEL-7704, SMMC-7721, Huh-7, and NB4 cells infected with miR-223 construct. The measurement of cell growth rate was performed by using CCK-8 kit. **P<0.01. N = 6. (F) IGF-1R expression was measured by quantitative PCR and was down-regulated in both SMMC-7721 and BEL-7404 cells infected with miR-223 construct. (G) After infection of miR-223 into Huh-7 cells, IGF-1R mRNA was also down regulated. All figures are representative of the study and additionally at least three independent experiments yielded similar results. *p<0.05.

To investigate IGF-1R as the general target of miRNA-223, miR-223 targeting IGF-1R was further studied in several other tumor cell lines. In NB4 cells, promyelocytic leukemia cells, which were treated with retinoic acid, the expression level of miR-223 increased abruptly (Fig. 8-A, left panel, *p*<0.05). While, IGF-1 mRNA expression was suppressed (Fig. 8-A, right panel) and the cell growth inhibited significantly with mature morphology change (Fig. 8-B). Transfection with miR-223 in NB4 cells also led to significant inhibition of IGF-1R mRNA and protein expression (*p*<0.05) (Fig. 8-C,D) and the cell growth (Fig. 8-E). In SMMC-7721, BEL-7404, or Huh-7 hepatoma cells infected with miR-223 constructs, all the cell growth rates slowed down (Fig. 8-E) and the expression level of IGF-1R was significantly inhibited (Fig. 8-F,G). This result suggested that miR-223 targeted IGF-1R not only in HeLa cells, but also in leukemia and hepatoma cells.

## Discussion

In this study, we established a miR-223 over-expression model and observed miR-223 suppression of cell growth, colony formation *in vitro*, and the tumorigenesis *in vivo* . These results suggest that miR-223 functioned as a negative regulator or tumor suppressor for the cell growth, which is consistent with the role of miR-223 in HCC [18]. In order to find out the mechanisms and target mRNAs that were responsible for the suppressive function of miR-223, we detected several putative targets of miR-223 including FBXW7, LMO2, STMN1, Mef 2C, NF1A and found that all their mRNA levels changed slightly. However, we observed that IGF-1R was strongly targeted by miR-223. There are several lines of evidence in our study to support this. First, IGF-1R 3̀UTR reporter experiment showed a significant decrease in luciferase activity after miR-223 over-expression. Second, mutation of three nucleotides of IGF-1R 3′UTR within miR-223 seed sequence binding site abolished the response to miR-223. When we inhibited miR-223 with synthetic sponge inhibitor, miR-223 lost its inhibitory function and the Renilla luciferase activities of IGF-1R 3′UTR reporter returned to the level similar to control group. Third, both mRNA and protein of IGF-1R were down regulated in miR-223 group as compared with EV group, but transfection with IGF-1R cDNA without 3′UTR sequence overcome the inhibition by miR-223 and rescued the expression of IGF-1R. These results strongly indicated that the wild type 3′UTR of IGF-1R interacted with miR-223 and was suppressed at both transcription and translation stages. Mature IGF-1R appeared at 2 bands which might be due to different modifications, but reduced together in miR-223 group.

IGF-1R signal pathway was involved in the development and tumorigenesis at many aspects. IGF signaling abnormality appears to directly interfere with the normal cell growth regulation and proapoptotic responses triggered by activation of p53, the tumor suppressor, upon the treatment with anti-cancer agents[22]. In this study, we investigated and found that PI3K/Akt/mTOR/p70S6K signal pathway was suppressed by miR-223 (Fig. 4-A). The signaling pathway of mTOR is important in miR-223 regulation of cell growth, which is consistent with the previous bioinformatics prediction [16]. We also noted that re-expression of IGF-1R in miR-223 group could abolish the inhibition effect of miR-223 and reversed the inhibition of IGF-1R-mediated downstream Akt/mTOR/p70S6K signal pathway. Further, knockdown of IGF-1R by shRNA could induce similar inhibitory effects with miR-223 on IGF-1R and Akt/mTOR/p70S6K signal pathway.

In the present study, miR-223 suppressed IGF-1R and its signaling and acted as a tumor suppressor. On the other hand, miR-223 is significantly up-regulated in bladder cancers [15], recurrent ovarian cancer [16] and increases cyclin E protein and activity levels, and elevates genomic instability [23]. Thus miR-223 might serve as a doubled-edge sword by targeting opposite functional targets, since one miRNA can target a dozen mRNAs which impact many molecules that are involved in different signal pathways. The dominant influence of a miRNA on the regulated function of cells may depend on the relative importance of the targets that are involved in different signal pathways. In this model, miR-223 targeted IGF-1R and its downstream signal pathway, which exerted a major function in tumor cell growth regulation. IGF-1R was the major mRNA among the miR-223 targets in our study. LMO2, STMN1, Mef 2C, FBXW7 and NF1A did not significantly decrease in our system. Indeed several other molecules including Rasa1 were also observed to be targeted by miR-223 in current study at both mRNA and protein levels. The luciferase reporter assay did show that the 3′UTR of Rasa1 gene was targeted by miR-223 directly. Rasa1 exerts a tumor suppressor function by removing GTP from RAS-GTP. Its down-regulation should activate the Rasa1/RAF/MEK/ERK signal pathway. However, ERK1/2 was down-regulated not only at total protein level, but also at phosphorylation level in miR-223 group as compared with the group, which indicated that ERK pathway was inhibited although Rasa1 was targeted. Rasa1 encodes p120-RasGAP — a RasGTPase which reverts active GTP-bound into inactive GDP-bound form [24]. Therefore Rasa1 could not be the functional target of miR-223 because it failed to regulate ERK pathway after miR-223 targeting. However, IGF-1R could be the functional target of miR-223, which was responsible for the inhibition of cell growth. Suppression of IGF-1R-mediated pathway might lead to inhibition of ERK signaling, which made Rasa1 lose its regulatory role in signaling. Furthermore, IGF-1R was also down regulated in NB4 (promyelocytic leukemia) cells infected with miR-223. When NB4 cells were induced with retinoic acid to differentiation, miR-223 was greatly up-regulated, but IGF-1R down regulated, which suggested that IGF-1R acted as the target. In hepatoma cells (SMMC-7721, BEL-7404, or Huh-7) IGF-1R also served as the common target when miR-223 inhibited the cell growth.

**Figure 9:**
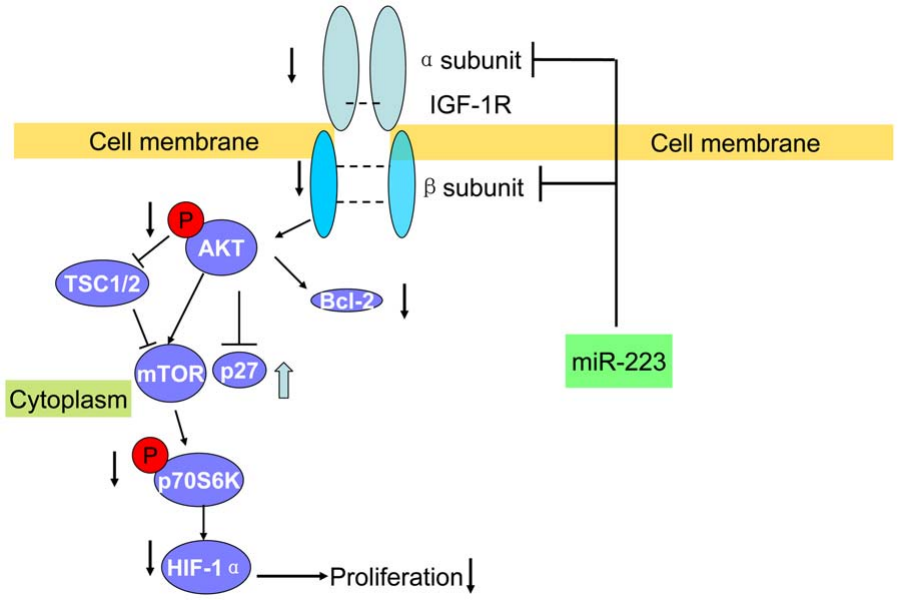
Summary of miR-223 inhibiting tumor growth by targeting IGF-1R and suppressing the downstream Akt/mTOR/p70S6K signal pathway. The arrow indicated the major results of up-regulation and down-regulation.

In summary (Fig. 9), we established a miR-223 overexpression model by using lentivirus delivery system and observed that miR-223 suppressed the proliferation of tumor cells both *in vivo* and *in vitro*. It was through IGF-1R and its downstream signal pathway that miR-223 suppressed the cell growth.

## Materials and Methods

### Plasmid construction

To construct the lentivirus vector pLL 3.7-miR-223 which expressed miR-223, a fragment encoding the pre-miR-223 sequence plus 110bp at both 5′- and 3′-flanking regions (chrX:65238602-65238931, from UCSC web site) was amplified with the primers 5′CCGGTTAACCTGGCAGTCCATTCGTCA3′and 5′CCGCTCGAGCCTCTAGGGTCACATCTCC3′ by PCR from NB4 cell genomic DNA and then cloned into the Hpa I/Xho l sites of pLL 3.7 vector.

Psi-*CHECK*™-2-IGF-3′UTR: The 3′ UTR fragment (3′untranslated region) of IGF-1R (Genbank ACCESSION: NM_000875) containing the binding site (from 1 to 3636 bp in the 3′UTR of IGF-1R) was amplified by PCR using the primers 5′CCCC**CTCGAG**GATCCTGAATCTGTGCAAAC and 3′AAAA**GCGGCCGC**CTTCCCAGCGAAATCATC 3′ and cloned into Xho I/Not I sites of psi-*CHECK*™-2. This vector allowed simultaneous expression of renilla and firefly luciferases. The IGF-1R 3′UTR was cloned downstream of the renilla luciferase gene allowing the expression of a renilla transcript with the 3′UTR from IGF-1R. Renilla luciferase activity was then used to assess the effect of the 3′UTR on transcript stability and translation efficiency. The second reporter, firefly luciferase serves as control.

Psi-*CHECK*™-2-IGF-3′UTR-mut: Three nucleotides of 3′UTR of IGF-1R within the perfect binding site with miR-223 seed sequence, was mutated at the position of 226-228, from AACTGAC to AAgacAC by PCR mutagenesis using the following primers 5′ AAACCCTTAA***GAC***ACATGGGCCT 3′ and 5′ AAGGCCCATGT***GTC***TTAAGGGTT 3′. Successful mutations were confirmed by DNA sequencing.

P*Silencer 4.1* CMV puro-IGF-1R-sh: To silence IGF-1R expression, we constructed a vector-based IGF-1R-shRNA to interfere the expression of IGF-1R. The oligonucleotides we used were 

5′GATCCGGCCAGAAATGGAGAATAATTCAAGAGATTATTCTCCATTTCTGGCCTCA3′ and 5′AGCTTGAGGCCAGAAATGGAGAATAATCTCTTGAATTATTCTCCATTTCTGGCCG3′ and were cloned into the Bam H1 and Hind III sites of p*Silencer-*4.1-CMV puro vector. The construction of IGF-1R-shRNA was confirmed by DNA sequencing.

### Cell culture, Lentivirus packaging and infection

HeLa, and HEK-293T cells from ATCC, SMMC-7721, BEL-7404, or Huh-7 cells from Biochemistry and Cell biology Institute of Shanghai, Chinese Academy of Science, were cultured in DMEM supplemented with 10% fetal calf serum (Gibco BRL, Carlsbad, CA, USA). The Lentivirus-mediated miR-223 packaging system contained three plasmids pLL3.7 or pLL3.7-miR-223, Δ8.9 and Vsvg at the ratio of 4∶3∶2 in quantity. Total of 12 µg of the plasmids were co-transfected with 30 µl Lipofectamine™2000 (Invitrogen, Carlsbad, CA, USA) into HEK-293T cells in a 100 mm diameter culture dish. When the transfected cells were at 80-90% confluence, the supernatant was collected 48 hours post-infection and filtered by a filter with 0.45 micrometer pore size and used as the virus source.

HeLa cells was infected with either pLL3.7(EV) or pLL3.7-miR-223 in the presence of 8 µg/ml polybrene (Sigma-Aldrich, St. Louis, MO, USA) for 12 hours, and the medium was refreshed. 72 hours post the infection, the efficiency of infection was measured under a fluorescent microscope and the cells were sorted by a FACS (fluorescent activated cell sorter, Becton Dickinson, Mountain View, CA, USA) based on the expression of GFP carried by pLL 3.7 plasmid. The sorted cells were taken as the over-expression model for the following experiments.

### Luciferase reporter assay

Luciferase reporter assay was performed in HEK-293T cells. Forty eight hours post transfection, the cells were washed with PBS twice and lyzed in 100 µl Passive Lysis Buffer (Promega, Madison, WI, USA) and the luciferase activities were measured from 20 µl lysate using the Dual Luciferase reporter assay kit (Promega, Madison, WI, USA) on a illuminometer (Lumat LB 9507, Berthold, Germany). All the data were obtained by averaging the results from six independent repeats. The mutated psi*CHECK*™-2-IGF-1R 3′UTR was also transfected under the same condition, and the miR-223 inhibitor and its control were used at the final concentration of 50 nM to measure the inhibitory effect of miR-223 on the 3′UTR of IGF-1R.

### Colony formation assay

The method was according to our previous report[25]. Briefly, cells were digested with trypsin and suspended into a single cell status. 6000 cells from each group were cultured in the 60 mm diameter culture dish with 10% FBS for 14 days. The colonies were fixed and stained with 0.5% crystal violet for 15 min, and then washed 3 times. The colonies consisting of more than 50 cells were defined as one colony. The number of colonies in 10 random view fields was counted under a microscope and the average representing the 95% confident region was achieved. The experiment was carried out 3 independent times.

### Tumor inoculation assay in nude mice

Female BALB/c athymic nude mice at the age of 5 to 6 weeks were from SINO-BRITISH SIPPR/BK LAB ANIMAL LTD, CO. (Shanghai, China). All the animals used in this study were approved by Shanghai Municipal Government with the permit number of 00800161308. All the procedures involving animals were according to the NIH Guide for the Care and Use of Laboratory Animals and local institutional ethical guidelines for animal experiment. This experiment was approved by Experimental Animal Ethics Committee, Fudan University Shanghai Medical College with the permit number of 20110307-092. 5×10^6^ miR-223 or EV-infected HeLa single cell suspensions in 150 µl sterile PBS were injected subcutaneously to the skin under the front legs of the mouse. Tumor growth was examined every 3 days for 5 weeks and its volume (V) was monitored by measuring the length (L) and width (W) of the tumor with calipers and calculated with the formula V =  1/2(L×W^2^).

### RNA extraction and quantitative PCR

Total RNA was extracted from cells using Trizol reagent (Invitrogen, Carlsbad, CA, USA) and reverse transcription reaction was performed with RT Kit according to the manufacturer's protocol (Invitrogen, Carlsbad, CA, USA) based on our previous report[26]. Normalization of miR-223 was performed by using RNU6 primers in miR-223 and β-actin in other protein coding genes, respectively. 50 nM miRNA specific stem-loop RT primers [27] were used for reverse transcription reaction as the following. 5′GTCGTATCCAGTGCAGGGTCCGAGGTATTCGCACTGGATACGACTGGGGT 3′ for miR-223 and 5′AAAATATGGAACGCT 3′ for the loading control RNU6, and quantitative PCR to detect mature miR-223 with specific primers was performed with 2× SYBR Green Master Mix (DBI Bioscience, Shanghai, China) according to the protocol as our previous report[28]. The primers used in this experiment are as the following: 5′GTGCAGGGTCCGAGGT3′ and 5′CGGGCTGTCAGTTTGTCA3′ for miR-223; 5′CTCGCTTCGGCAGCACA 3′ and 5′AACGCTTCACGAATTTGCGT 3′ for RNU6; …5′GAGCTACGAGCTGCCTGACG3′ and 5′CCTAGAAGCATTTGCGGTGG3′ for loading control β-actin; 5′TGAACAGAATGGAATGGAGC3′and 5′ACTTTCATCCATTGATTGCC3′ for HIF-1α; 5′TTATGTTCTTTTTCCCCCTT3′ and 5′ TGCCCACAAATTATCTTCTATC3′ for p70S6K; 5′ GGACAGGTCAGAGGGTTTC and 5′CTCGTAACTCTTCTCTGTGCC3′ for IGF-1R-1; 5′ATTCCAGCAGTCCCAGTTAT3′ and 5′CATCTCCCAATGCTGCTTAG3′ for IGF-1R-2; 5′ GCAACCGACGATTCTTCTAC3′ and 5′TCGAGCTGTTTACGTTTGAC3′ for p27; 5′TACTACCGCCTCACACGC3′ and 5′CTCCTCCTCCTCCTCTTCCT 3′ for cyclin D1; 5′GGCTTCAGAAATACTTCCACC3′ and 5′ GCAGGACAGATGAGTCGAAG 3′for Rasa1; 5′ TGGGACTTGAGTGGTTTGTAG3′ and 5′CAGTTTAGGTTGCTACAGGCT3′ for Fox O1; 5′CTGAGGAAGGGGAAGTGG3′ and 5′ GTAAACGGTATCACTGTCCACT3′ for Fox O3; 5′ ACATTAGTGGGACATACAGGTG3′ and 5′CATACAACGCACAGTGGAAGT3′ for FBXW7. PCR was performed by iQ5™ quantitative PCR detection system (Bio-Rad, Alfred Nobel Drive, Hercules, CA, USA) and results were analyzed with IQ5 software. All reactions were run in triplicate and all experiments were carried 3 independent times.

### Western blot

Fifty micrograms of total proteins were loaded onto 10% sodium dodecyl sulfate–polyacrylamide gel electrophoresis and transferred onto polyvinylidene difluoride(PVDF) membranes (Millipore, Billerico, Massochusatts, USA). After blocking with 1% bovine serum albumin, the blots were incubated with antibodies against IGF-1R, HIF-1α, p70S6K, p- p70S6K, Akt, p-Akt (Bioworld, Atlanta, Georgia 30305, USA), p27, cyclin D1(Neomarkers, Fremont, CA, USA), ERK1/2, p-ERK1/2, or Bcl-2(Cell Signaling Technology Inc, 3 Trask Lane, Danvers, MA 01923, USA) glyceraldehyde- 3-phosphate dehydrogenase(GAPDH) (Chemicon International, Temecula, CA, USA). After incubation with horseradish peroxidase-conjugated secondary antibody, protein bands were visualized using enhanced chemiluminescence detection kit (Millipore, Billerico, Massochusatts, USA). The intensity of the bands was analyzed using Image-Pro Plus software.

### Immunohistochemistry

Immunohistochemistry was performed according to our previous documents [29], [30]. Tumor specimens were taken from the sacrificed nude mice, dehydrated with 15%/30% gradient sucrose, and fixed with opti-mum cutting temperature compound (OCT)(SAKURA, Tokyo, Japan). The sections were permeabilized with 0.5% Triton-X100 at room temperature for 10 min, washed three times with PBS, blocked with 5% donkey serum, and incubated with IGF-1R antibody in 5% donkey serum at 4°C for 24 hours. The sections were washed and incubated with secondary antibody (lexa Fluor 594-conjugated donkey anti-rabbit IgG) (Invitrogen, Carlsbad, CA, USA). The sections were then rinsed with PBS and examined under a fluorescent microscope.

### Proliferation and interference experiment

Logarithmic growth phase HeLa cells were digested with trypsin to single cell suspension and 3000 cells were transferred to each well of a 96-well plate. CCK-8 (Cell Counting Kit-8) reagent was added at the time point of 12, 24, 48, 72, 96, and 120 hours after seeding and incubated at 37°C for half to 4 hours according to the color change. The data of OD (optical density) value at 450 nm were read by a microplate reader. Each experiment was performed 3 times independently.

### Wright-Giemsa stain

The staining procedure was based on our previous reports [9], [10]. Cell smears were freshly prepared and dried in open air. Wright-Giemsa stain solution was placed on the slide to cover all the cells and incubated for 10 min. The slide was washed and rinsed with distill water. The slide was viewed under an Olympus microscope.
